# Cerebellar Functions Beyond Movement and Learning

**DOI:** 10.1146/annurev-neuro-100423-104943

**Published:** 2024-07-01

**Authors:** Linda H. Kim, Detlef H. Heck, Roy V. Sillitoe

**Affiliations:** 1 Department of Pathology & Immunology, Baylor College of Medicine, Houston, Texas, USA; 2 Jan and Dan Duncan Neurological Research Institute, Texas Children's Hospital, Houston, Texas, USA; 3 Department of Biomedical Sciences, University of Minnesota Medical School, Duluth, Minnesota, USA; 4 Center for Cerebellar Network Structure and Function in Health and Disease, University of Minnesota, Duluth, Minnesota, USA; 5 Departments of Neuroscience and Pediatrics, Program in Developmental Biology, and Development, Disease Models & Therapeutics Graduate Program, Baylor College of Medicine, Houston, Texas, USA

**Keywords:** cerebellum, movement, learning, cognition, reward, sleep

## Abstract

The cerebellum has a well-established role in controlling motor functions, including coordination, posture, and the learning of skilled movements. The mechanisms for how it carries out motor behavior remain under intense investigation. Interestingly though, in recent years the mechanisms of cerebellar function have faced additional scrutiny since nonmotor behaviors may also be controlled by the cerebellum. With such complexity arising, there is now a pressing need to better understand how cerebellar structure, function, and behavior intersect to influence behaviors that are dynamically called upon as an animal experiences its environment. Here, we discuss recent experimental work that frames possible neural mechanisms for how the cerebellum shapes disparate behaviors and why its dysfunction is catastrophic in hereditary and acquired conditions—both motor and nonmotor. For these reasons, the cerebellum might be the ideal therapeutic target.

## 1. INTRODUCTION

The cerebellum is well recognized for its vital role in controlling motor coordination and learning (Lang et al. 2017, Manto et al. 2012). However, with recent advances in experimental tools, there is now a wealth of evidence that profoundly expands our understanding of the cerebellum toward the regulation of various nonmotor behaviors, including cognition, memory, language, mood, and sleep (Adamaszek et al. 2017, Baumann et al. 2015, Koziol et al. 2014, Mariën et al. 2014, McAfee et al. 2021, Popa & Ebner 2018, Schmahmann et al. 2019, Van Overwalle et al. 2020). Accordingly, a growing list of cerebellar abnormalities (structural and functional) have been linked to the clinical manifestations of both motor and nonmotor diseases (Bareš et al. 2019, Haldipur et al. 2022, Olivito et al. 2023, Sokolov et al. 2017). The unique functional characteristics of the cerebellum, in terms of processing capabilities and inherent capacity for different forms of plasticity, make it an appealing therapeutic target. These potential benefits are also reflected in its anatomy. Despite occupying only 10% of the brain's volume, the cerebellum houses a remarkable 80% of the brain's neurons (Azevedo et al. 2009, Herculano-Houzel et al. 2015). This compactness is achieved by densely packing the cells in a columnar arrangement, forming modules that are perpendicular to the cortical surface and parallel to one another (Apps & Hawkes 2009, Cerminara et al. 2015). This modular organization, along with extensive cerebellar interconnections with the forebrain and brainstem, gives the cerebellum the foundation for parallel processing that can support the rapid adaptive control of the body in various environments and across diverse behaviors. Adaptive behavior and its mechanisms are often categorized into separate domains, including sensory, motor, cognitive, language, and memory. However, the brain functions as a unified whole. Therefore, it is vital to grasp how the cerebellum, central in processing predictions that shape neural representations of context-specific dynamics for adaptive control, integrates these diverse functions across multiple domains.

This review highlights recent experimental work that provides a framework for understanding how cerebellar structure, circuitry, and function intersect to dynamically shape various behaviors. Our goal is to discuss the broadening scope of cerebellar-dependent nonmotor behaviors, as well as to consider the implications of its dysfunction in neurological and neuropsychiatric diseases. With this goal in mind, we

1. Introduce the key features that define cerebellar structure, connectivity, and its regional heterogeneity;
2. Discuss the role of the cerebellum in nonmotor behaviors, although we use the knowledge from its roles in motor behavior to argue about the possible mechanisms;
3. Highlight cerebellar involvement in neurological and neuropsychiatric diseases; and
4. Theorize about the potential neural mechanisms that allow the cerebellum to support various behaviors in the context of its dysfunction in diverse diseases.

## 2. STRUCTURE OF THE CEREBELLUM

### 2.1. Cellular Composition and Connectivity

**Figure 1 (:**
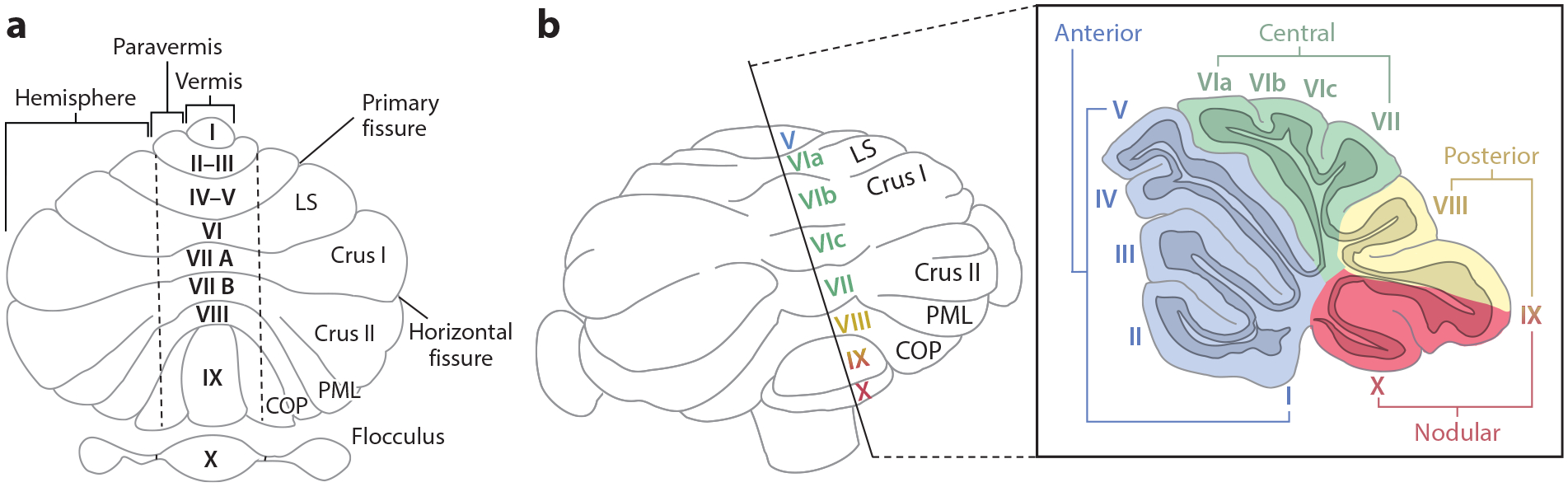
*a*) A schematic of a flattened cerebellum in human showing the three longitudinal compartments (the vermis, the paravermis, and the hemispheres) along the mediolateral axis and the 10 evolutionarily conserved lobules. The primary fissure divides the anterior (lobules I–V) and posterior (lobules VI–X) lobes. (*b*) A schematic of a rat cerebellum shown from a dorsal view (*left*) with a sagittal section of the vermis (*right*) illustrating the four transverse domains with separate colors. Note that cerebellar lobule VIc is clearly represented in certain rodents and higher mammals. Abbreviations: COP, copula pyramidis; LS, lobulus simplex; PML, paramedian lobule. Figure adapted from illustration by Megan X.V. Nguyen.

The cerebellum can be partitioned into different anatomical units based on its different axes. From external to internal, the outer shell of the cerebellum is called the cerebellar cortex, which because of its three-dimensional architecture completely covers the cerebellar nuclei (CN) that are housed at its core. The cerebellum's prominent folded appearance features fissures that divide the cortex into lobules along the anterior-posterior axis (Figure 1). These folds are distinct in their partitioning from three broad anatomical regions that span the mediolateral axis: the vermis (at the midline), the paravermis (adjacent to the midline), and the hemispheres (the lateral regions). The hemispheres are the largest cerebellar region in mammals, who also have a further anatomical distinction of the paraflocculi and flocculi, which extend laterally and curl beneath the cerebellum.

#### 2.1.1. Internal cerebellar circuitry.

**Figure 2:**
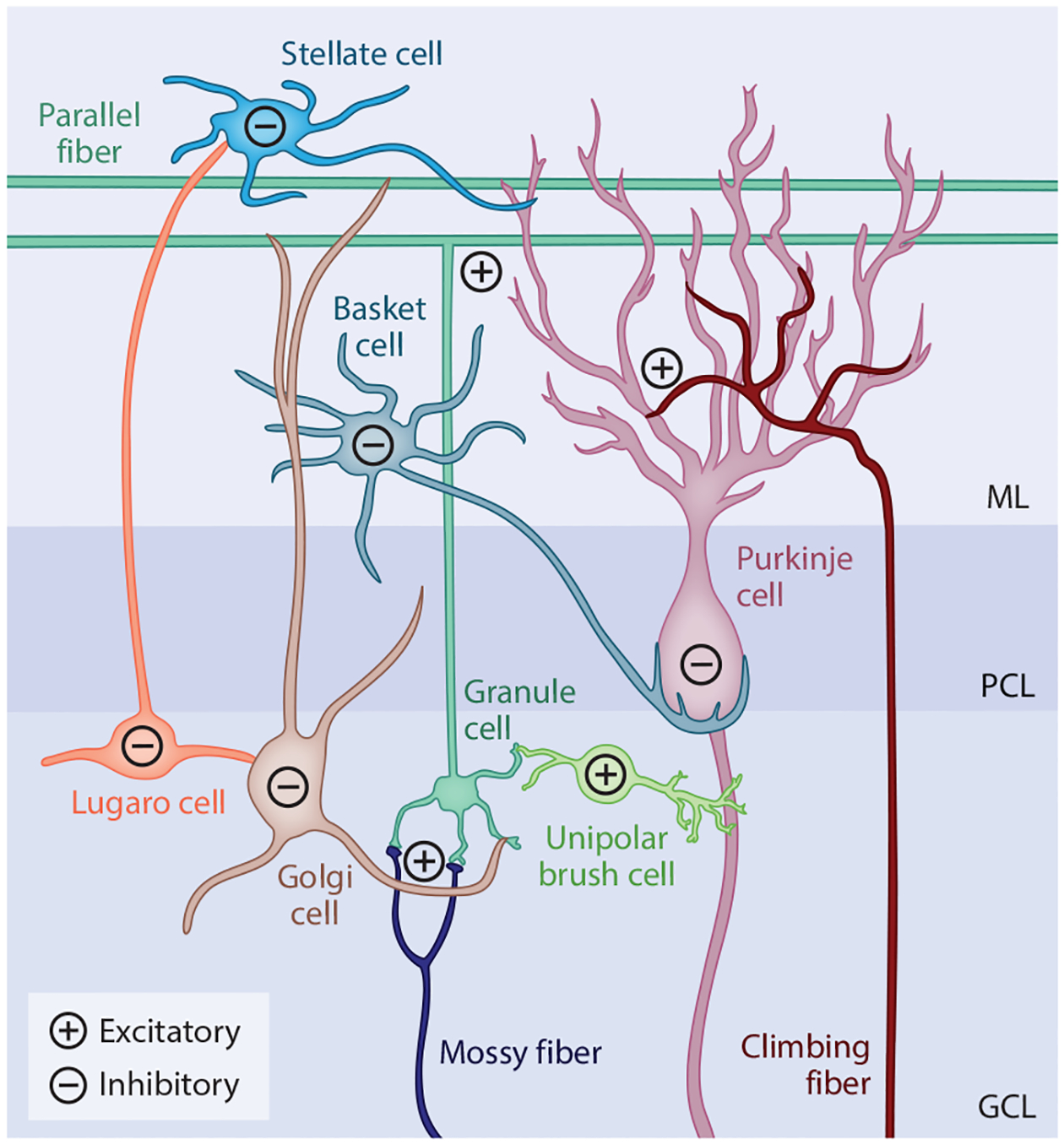
The cytoarchitecture and neuronal circuitry of the cerebellar cortex are organized into three distinct layers: the molecular layer (ML), the Purkinje cell layer (PCL), and the granule cell layer (GCL). Mossy fibers and climbing fibers provide the major excitatory afferents into the cerebellum. The ML contains inhibitory interneurons (stellate and basket cells) as well as excitatory parallel fibers and climbing fibers that carry input signals to the Purkinje cell dendrites. Climbing fibers originate in the inferior olive, whereas parallel fibers are the transverse portions of the axons of the granule cells. The PCL is mainly composed of Purkinje cell somata. The Purkinje cells are the sole output neurons of the cerebellar cortex and send inhibitory projections onto the cerebellar and vestibular nuclei neurons. The GCL contains the excitatory granule cells and unipolar brush cells as well as inhibitory Golgi cells and Lugaro cells. Other afferents such as beaded fibers and cell types that include candelabrum cells and Bergmann glia are not shown for simplicity. Figure adapted from illustration by Megan X.V. Nguyen.

The cerebellar cortex contains three distinct layers: the molecular layer (superficial), the Purkinje cell (PC) layer (middle), and the granular layer (innermost) (Figure 2). The molecular layer contains inhibitory basket cells and stellate cells [collectively called the molecular-layer interneurons (MLIs)], as well as excitatory parallel fibers (PFs) and climbing fibers (CFs) that deliver sensorimotor signals to the PC dendrites. The axons of granule cells (GrCs) originate in the granular layer, project vertically, and then bifurcate into PFs that extend transversely within the molecular layer to form excitatory synapses on PC dendritic spines. Each PF can synapse with hundreds of PCs. PCs perform the main computations in cerebellar information processing, with each cell receiving more than 200,000 inputs in mammals. The PC layer contains a monolayer of PC somata, Bergmann glia, and candelabrum cells. The granular layer contains GrCs, terminals of mossy fibers (MFs), and unipolar brush cells that excite GrCs. Each MF forms synapses with hundreds of GrCs. The GrCs also receive inhibitory inputs from local interneurons such as Golgi cells. Altogether, the activity of the sole output neurons of the cerebellar cortex, PCs, is regulated by excitatory inputs from PFs and CFs and inhibitory inputs from MLIs. The PCs send inhibitory GABAergic projections onto neurons in the CN, which contain glutamatergic, glycinergic, and GABAergic neurons that transmit the final output of the cerebellum to the rest of the brain and the spinal cord. The CN neurons also receive glutamatergic excitatory inputs from the collateral branches of MFs and CFs (Sugihara 2011, Thanawalla et al. 2020).

#### 2.1.2. Inputs and outputs.

There are three major afferent pathways to the cerebellum: (*a*) the CFs originating from the contralateral inferior olive; (*b*) the MFs originating from the spinal cord, pontine nuclei, brainstem reticular formation, and vestibular nuclei (among other regions); and (*c*) thin beaded fibers originating from several regions of the brain, including the locus coeruleus and raphe nucleus that terminate in all layers of the cerebellar cortex and the CN. Synaptic in vivo activity from MFs to PCs (via PFs) leads to high-frequency firing of simple spikes (SSs, ~65 Hz) in the PCs, whereas CF activity leads to more infrequent responses called complex spikes (CSs, ~1 Hz).

In primates, the CN are subdivided from medial to lateral into the fastigial, emboliform, globose, and dentate nuclei; in rodents, they are fastigial, interposed, and dentate nuclei, with each receiving inputs from different regions of the cerebellar cortex and sending back different patterns of axonal projections to the brainstem or the cerebral cortex via the thalamus (Buisseret-Delmas & Angaut 1993, Fujita et al. 2020, Teune et al. 2000). The fastigial nuclei receive afferents from the vermis and project to the vestibular nuclei, reticular formation, and vestibulospinal and reticulospinal tracts. The interposed nuclei receive input from the paravermis and project to the contralateral red nucleus and the thalamus. The dentate nuclei are the most lateral; they receive afferents from the hemispheres and project to various cortical and subcortical regions, including the contralateral red nucleus and the ventrolateral thalamic nucleus. Broadly speaking, the vermal and paramedian areas are mainly associated with sensorimotor behaviors, whereas the hemispheres are linked to nonmotor behaviors (for a review, see Cerminara et al. 2015). Although the regional heterogeneity of the cerebellum as it relates to behavioral specialization is far from clear, it is likely superimposed and integrated into the different architectural, molecular, and functional maps in the cerebellum.

In general, distinct output channels of the CN eventually loop back onto different cerebellar cortical areas, forming closed-loop cerebro-cerebellar interactions that modulate corresponding sensorimotor processes (Apps et al. 2018, Ruigrok et al. 2015, Voogd 2011). Note though that the number of reciprocal connections can vary across regions (Houck & Person 2014). Moreover, MF inputs from multiple modalities can also converge onto a single GrC (Chabrol et al. 2015, Huang et al. 2013, Ishikawa et al. 2015, Shimuta et al. 2020). Anterograde mono-transsynaptic tracing shows that MF terminals, which originate from the primary motor, sensory, and association cortical areas, spatially overlap within molecularly defined cerebellar modules of crus I, paraflocculus, and vermal regions IV/V and VI, suggesting the presence of cerebellar hubs with multimodal cortical influence (Henschke & Pakan 2020). Executive processes and rapid adaptive behavior in various environments may require the harmonious coordination between sensory-motor and cognitive cerebellar regions, assuming that there is some distinction between these regions.

### 2.2. Cerebellar Regional Heterogeneity

While the cerebellum displays a highly stereotyped anatomical organization, profound flexibility in its circuit organization and function is supported by molecular expression patterns and region specificity (see the Supplemental Material section titled Cerebellar Zonal Organization). The pattern of stripes can be used to segment the cerebellum into four transverse zones that each receive functionally distinct afferent fibers and have a unique complement of stripes: anterior (lobules I–V), central (lobules VI–VII), posterior (lobules VIII–IX), and nodular (lobules IX–X) zones (Ozol et al. 1999) (Figure 1b). Broadly, motor regions lie in the anterior and posterior zones as well as portions of the central zone, whereas nonmotor regions encompass lobule VI, crus I, crus II, lobule VIIB, and the nodular zone. However, one should be careful with such generalization as lobular boundaries and zones do not perfectly reflect functional boundaries (Apps & Hawkes 2009, King et al. 2019).

**Figure 3:**
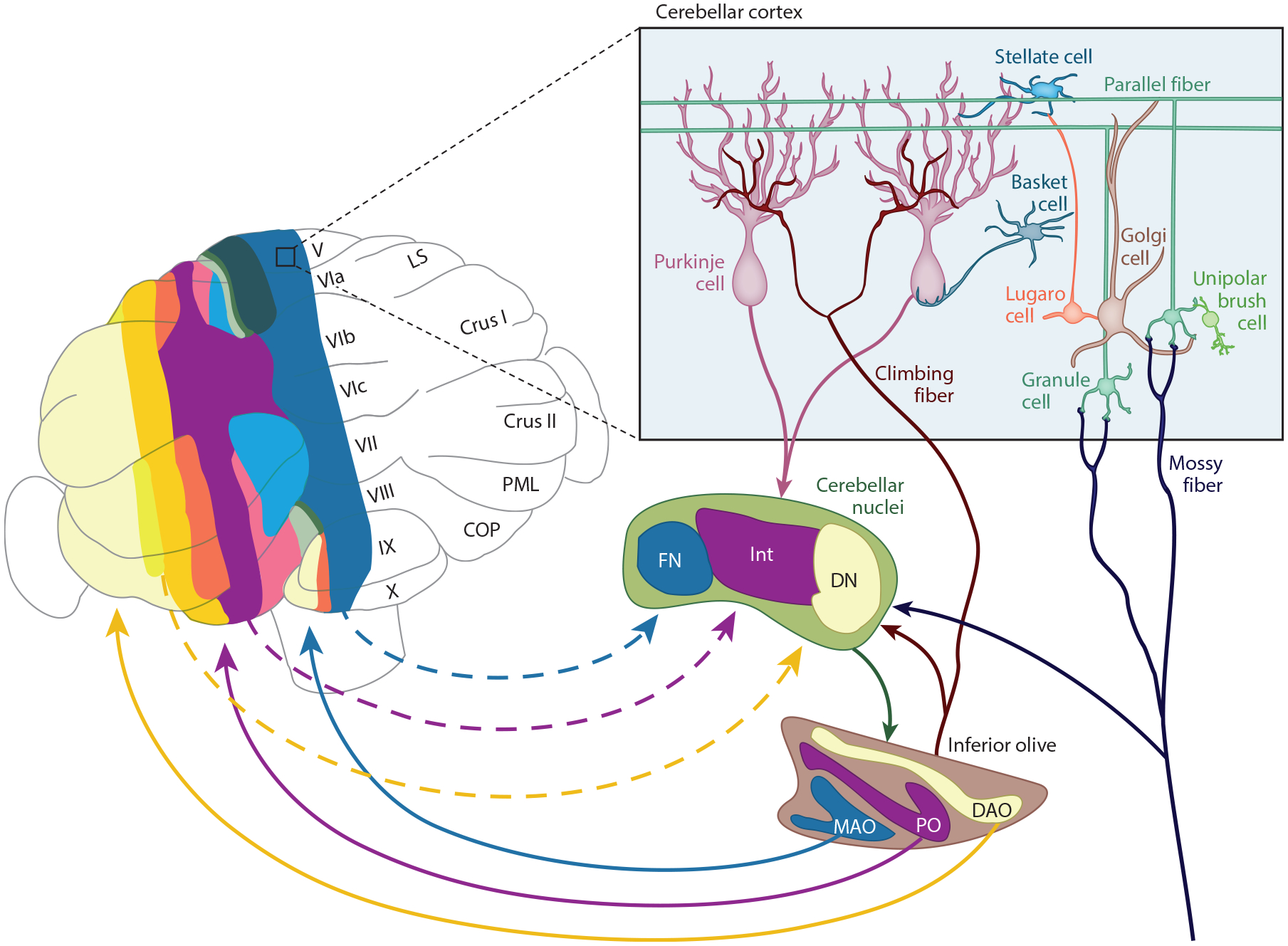
An overview of olivo-cortico-nuclear loops in the cerebellum with an example of a cerebellar cortical microcircuit. The zonal organization is depicted in different colors. Abbreviations: COP, copula pyramidis; DAO, dorsal accessory olive; DN, dentate nucleus; FN, fastigial nucleus; Int, interposed nucleus; LS, lobulus simplex; MAO, medial accessory olive; PML, paramedian lobule; PO, principal olive. Figure adapted from illustration by Megan X.V. Nguyen.

Recent studies have revealed multiple recurrent feedback loops and substantial molecular heterogeneity in the cerebellum (for a review, see Kebschull et al. 2024), prompting a reevaluation of our comprehension of cerebellar function. Thus, a complete depiction of the intricacies of cerebellar anatomical and functional connectivity and the specific impact on regional processing remains elusive, which is in part due to the complex organization and diverse dynamics of afferent and efferent connections. PC projections to the CN are topographically organized into rostrocaudal domains, forming longitudinal zones of PCs, and therefore contribute to forming the cerebellar modules (Apps & Hawkes 2009) (Figure 3; see also the Supplemental Material section titled Cerebellar Modular Organization). While the modules are traditionally thought to follow mono-somatotopic organizations (Manni & Petrosini 2004) that get linked up by PFs for complex behavior (Thach et al. 1992), there is complexity to note. For instance, the rostral anterior interposed nuclei contain specialized modules for coordinating stereotyped poly-somatotopic motor synergies such as defensive reflexes (Heiney et al. 2021). The rostral anterior interposed nuclei neurons receive predominantly mono-somatotopic PC inputs; and yet, they demonstrate a poly-somatotopic input inhibition and output capabilities that integrate sensory and motor signals from various body parts (Heiney et al. 2021). Given that there are also specialized modules for reducing complexity and enhancing precision of a single body part in multijointed movements such as reaching (Becker & Person 2019), conventional mono-somatotopic body maps in the cerebellum may coexist with ethologically relevant behavior-based maps, as seen in the motor cortex (Graziano 2016).

## 3. CEREBELLAR FUNCTION IN BEHAVIOR

Many motivated behaviors belong on a continuum of dichotomized or bivalent states: approach-avoidance, appetitive-aversive, proactive-reactive, convergent-divergent, and so on. This bivalence in behaviors has been central to neural evolution (Cisek 2022), suggesting that the cerebellum evolved under pressure to have the neural capacity for rapid error predictions and adaptive control of the body when specific demands in various environments arise. Despite the cerebellum's highly stereotyped organization and its relatively small number of well-documented cell types, the cerebellar circuit exhibits remarkable heterogeneity that can increase the flexibility of its circuit organization and function. Thus, it is ideally built to support specialized functions starting from the segregation of functions involving molecular, cellular, or local network specialization and the distribution of functions that converge across numerous cerebellar lobules to interact with the forebrain. The modular organization of the cerebellum and the microcircuits that comprise them may allow it to compound or co-opt its circuits to accommodate parallel processing of various demands for diverse tasks. This has long inspired the overarching hypothesis that basic cerebellar computational principles are conserved across motor and nonmotor domains. Therefore, we first turn to the motor function literature for insights before discussing cerebellar nonmotor functions.

### 3.1. Cerebellar Motor Function

The cerebellum is critical for fine motor control, motor coordination, and learning new movements that require precision and accurate timing. The Marr-Albus-Ito theory (Albus 1971, Ito 1972, Marr 1969) is a widely accepted framework that outlines the computational principles underlying the cerebellum's ability to perform error-based learning and internal modeling to refine and improve motor performance. Rooted in this framework is the hypothesis that the cerebellar cortex is organized into repeated modules that are thought to differentiate incoming sensorimotor information and then selectively modify the motor output. As an example of the theory's applicability, even within the confinement of a single movement around the same axis in space, photoactivating subpopulations of PCs promote distinct kinematic profiles (Blot et al. 2023).

#### 3.1.1. Error-based learning.

Error-based cerebellar learning was originally focused on bidirectional synaptic plasticity at PF-PC synapses, where long-term depression (LTD) is controlled by the absence of CF input and long-term potentiation (LTP) is driven by the presence of CF input plus PF activity (for a review, see Kawato et al. 2021). However, various types of synapses in the granular and molecular layers have also demonstrated plasticity (for reviews, see Gao et al. 2012, Hull & Regehr 2022). PCs were thought to make kinematic predictions about ongoing movements via high-frequency SSs and receive sensory error information about that movement via low-frequency CSs. However, the interaction between SS and CS activity compels us to reconsider this traditional perspective (for reviews, see De Zeeuw 2021, Streng et al. 2022). Following PC activity in the oculomotor vermis of animals during saccades, error direction influences the probability of CSs, while error magnitude alters their temporal distribution (Herzfeld et al. 2018). SSs also convey substantial predictive and feedback signals related to kinematics or position errors and are dynamically modulated by CSs (Streng et al. 2017, 2022). Moreover, LTD more likely occurs in regions with higher spike activity, whereas lower firing frequencies offer greater potential for LTP. Thus, zebrin-positive PCs with lower spontaneous firing can be readily enhanced (i.e., upbound), while zebrin-negative PCs with higher spontaneous activity are more susceptible to LTD (i.e., downbound) (De Zeeuw 2021, Wu et al. 2019). PF synaptic plasticity in PCs and MLIs is influenced by CF activity and is bidirectional and reciprocal (Badura et al. 2013, Gao et al. 2012, Jörntell & Ekerot 2002). As CF activity diminishes, the PCs are prone to experiencing both LTP at the PF-PC synapse and LTD at PF-MLI and MLI-PC synapses. Then, as CF activity increases, the PCs are prone to LTD at the PF-PC synapse and LTP at PF-MLI and MLI-PC synapses. Spatial differences in where a learning mechanism might dominate can be observed if one considers the locations of zebrin-positive versus zebrin-negative regions, such as the flocculus of the vestibulocerebellum for vestibulo-ocular reflex adaptation and hemisphere lobule VI for classical eyelid conditioning, respectively (see the Supplemental Material section titled Differences in Learning Mechanisms).

#### 3.1.2. Internal models and cerebellar function.

Error-based learning and internal modeling are closely intertwined and iterative. Internal modeling involves the formation and the application of internal representations of the body's dynamics and the environment within the cerebellum. Predictions based on the sensory consequences of motor commands form forward models, and predictions of motor commands that are necessary for a goal generate an inverse model (Wolpert et al. 1998). While there is evidence to suggest that the cerebellum might incorporate certain aspects of inverse modeling under specific circumstances, depending on available error information (Ito 2013), the prevailing consensus is that its main role is forward prediction for voluntary limb movements or other nonmotor cognitive functions (for a review, see Tanaka et al. 2020). Detecting errors by comparing predicted signals with actual signals allows the cerebellum to continuously refine and update its internal models and rapidly generate accurate and adaptive coordinated motor commands (Bastian 2006, Popa & Ebner 2018, Shadmehr et al. 2010, Streng et al. 2018). Imaging studies show robust cerebellar activation in response to sensory prediction errors (Schlerf et al. 2012) and task performance errors (Diedrichsen et al. 2005, Flament et al. 1996, Grafton et al. 2008). Furthermore, reward processing in the cerebellum is thought to involve reward expectation driven by GrCs and reward prediction error driven by CFs. These nodes reciprocally modulate brain-wide reward circuitry and expand error-based cerebellar learning to reinforcement learning driven by reward and movement successes (for reviews, see De Zeeuw et al. 2021, Kawato et al. 2021, Wagner & Luo 2020).

#### 3.1.3. Motor representations and memory.

The exact mechanism by which cerebellar cortical synapses encode and retain complex adaptive representations remains enigmatic (Gao et al. 2012). Various types of plasticity within the granular layer and molecular layer occur in synergy and are distributed across time and space (D'Angelo et al. 2016, De Zeeuw 2021, Gao et al. 2012). By analyzing the synaptic connectivity maps between GrCs and PCs through whole-cell recordings with glutamate uncaging, it has been observed that individual mice display individualized combinations of functional connectivity traits that are associated with their specific locomotor activities (Spaeth et al. 2022). Moreover, these maps undergo acute modifications during postnatal development and under diverse adaptive locomotor conditions, influenced by somatotopic hardwiring but not deterministically constrained by it. Recent physiological and computational studies have additionally unveiled an intricate array of cerebellar neuronal and synaptic properties for producing the required input-output transformation and plastic changes, which involves complex spatiotemporal transformation of MF inputs and leveraging CF inputs for plasticity (for a review, see De Schepper et al. 2022). Further efforts to connect the intrinsic properties of different modules and their impact on encoding strategies, alongside the broader mapping of anatomical subdivisions and their role in encoding and retaining behaviors, are crucial for grasping how the cerebellum encodes and retains its rapid adaptive control of the body and limbs in various environments and across diverse complex behaviors.

### 3.2. Cerebellar Nonmotor Functions

Adaptive behavior requires both movement and cognition. Without action, there is no support for thought, and conversely, thought guides action. So how does cerebellar structure and function facilitate this diversity in adaptive behaviors that span both motor and nonmotor domains? There are at least three viewpoints regarding this matter. One views the cerebellum as a prediction machine that generates internal models that mimic the fundamental characteristics of cognitive representations found within the cerebral cortex and employs similar error-based learning for sensorimotor functions (for reviews, see Ito 2008, Olivito et al. 2023). Another perspective is that cerebellar activation reflects the use of imagery that co-opts representations and processes that are engaged during actual movement or cognition. Indeed, the cerebellum is active during imagined finger tapping (Hanakawa et al. 2008) and inner speech (Ackermann et al. 1998). Moreover, the current state of research suggests the view that the cerebellum is critical for event timing, specifically linked to discrete isolated intervals rather than continuous task dynamics (Liu et al. 2020), spanning both motor and perceptual contexts and implicit or explicit timing tasks (Bareš et al. 2019). Evidence for these three viewpoints can be readily found, especially since they are inter-related to some extent. Although these perspectives reveal common mechanisms for both motor and nonmotor functions, below we emphasize the specialized circuit and information processing adaptations that support cerebellar involvement in higher-order nonmotor functions.

Functional imaging studies in humans have unveiled cerebellar engagement across different tasks involving language, working memory, and emotional and social processing (Damasio et al. 2000; Guell et al. 2018; King et al. 2019; Ploghaus et al. 1999, 2000; Stoodley et al. 2012; Tedesco et al. 2011). Based on these and other works, there is now an increased recognition for cerebellar involvement in various higher-order nonmotor functions (for reviews, see Adamaszek et al. 2017, Clausi et al. 2022, Heck et al. 2023, Strick et al. 2009, Van Overwalle et al. 2020). This growing understanding is supported by neuroanatomical data revealing substantial connections between the cerebellum and cerebral cortex association regions, particularly with the prefrontal cortex (PFC) and limbic system, shedding light on the observed cognitive deficits in individuals with a cerebellar damage (for reviews, see Bodranghien et al. 2016, Ciapponi et al. 2023, Koziol et al. 2014).

Recent studies have identified monosynaptic glutamatergic projections from the CN to the ventral tegmental area (VTA) (Baek et al. 2022, Beier et al. 2015, Carta et al. 2019) that, upon optogenetic activation, can modulate reward processing and social behavior during place preference and social interaction tests, respectively (Carta et al. 2019). Furthermore, chemogenetic activation of crus I PCs that send synaptic inputs to VTA-projecting CN neurons contributes to stress-dependent development of depression-like behaviors (Baek et al. 2022). In a different behavioral context, fear conditioning is known to involve the amygdala and other interconnected brain regions such as the hippocampus, PFC, and periaqueductal gray. However, there is substantial evidence that suggests that the cerebellum plays a critical role as well (for a review, see Doubliez et al. 2023). Fear conditioning is a learning process by which an association is formed between a neutral stimulus and an unconditioned stimulus, such as an aversive foot shock, to elicit unconditioned responses such as freezing and escape behavior. LTP at PF-PC synapses in vermal lobules V and VI, but not in lobules IX and X, is critical for recalling cued fear memories (Sacchetti et al. 2004). Furthermore, photoactivation of monosynaptic glutamatergic projections from the CN to the lateral parabrachial nucleus evokes conditioned freezing responses, and photoinhibition impairs auditory fear memory (Hwang et al. 2023). While the eyelid conditioning paradigm offers key insights into learning, the fear conditioning paradigm necessitates nonmotor elements beyond the immediate motor reflex seen in eyelid conditioning and may inform about mechanisms that are distinct from or in addition to those in eyelid conditioning. Presumably, more anatomical subdivisions will be involved in coordinating both motor and nonmotor components that may require higher multimodal input-output integration, differential encoding and plasticity mechanisms, and differential tuning of local microcircuit properties depending on the scope of coordination. Recently, the influence of the cerebellum on a brain-wide network during multiday flexible behavior has been demonstrated, where chemogenetic inhibition of lobule VI and crus I PCs reduced coordinated activity within the thalamic subregions and between sensorimotor and associative subnetworks, respectively (Verpeut et al. 2023). We delve deeper into cerebellar contributions to higher-order processing in subsequent sections, focusing on two cognitive processes that are supported by interactions between the cerebellum and forebrain.

#### 3.2.1. Cerebellar contribution to spatial working memory.

Spatial working memory (SWM) refers to the ability to temporarily hold and manipulate spatial information in one's mind for the purpose of guiding behavior. Such tasks require interactions between different brain areas, forming temporary networks that require coordinated changes in functional connectivity between them (Aertsen et al. 1989, Vaadia et al. 1995). The thalamus plays a crucial role in facilitating coherence and synchrony between the cerebellum and the cortex in a task-dependent manner (for reviews, see Habas et al. 2019, Heck et al. 2023, Jones 2001, Nieuwhof et al. 2022). Coherence is a measure of the stability of phase relations between two oscillations of similar frequencies. The communication through coherence theory, initially proposed by Fries (2009), suggests that coherent oscillatory activities between different brain regions facilitate communication and coordination. Concurrent recordings in the medial PFC and the hippocampus during SWM tasks show increased coherence of theta oscillations during the decision process that reaches higher values with correct decisions than with incorrect decisions (Hyman et al. 2010, Jones & Wilson 2005, Liu et al. 2022). Importantly, photoactivation of PCs in the lobulus simplex disrupts decision-related coherence increases and impairs SWM, suggesting causal cerebellar involvement in optimizing task-related coherence and enhancing neuronal communication in a task-related manner (Liu et al. 2022). The anatomical circuits that mediate this behavior are still unclear. However, recent comprehensive tracing studies have unveiled extensive connections across all three CN divisions and the thalamic nuclei (Bohne et al. 2019, Fujita et al. 2020, Pisano et al. 2021); but each of these pathways could potentially have distinct physiological effects on cerebellothalamocortical coherence due to substantial pathway-specific differences in postsynaptic strength and neurotransmitter types (Bjerke et al. 2021, Gornati et al. 2018). Further investigations are required to resolve these issues.

#### 3.2.2. Cerebellar contribution to sleep.

Sleep research is vital due to its profound impact on health, cognition, and well-being, and yet, the contribution of the cerebellum to sleep has largely been neglected even though its influence on this behavior is accepted at multiple levels. The circadian timing system, consisting of a network of cerebral clocks and peripheral oscillators, is crucial in regulating daily cycles of physiological behavior, including regulating sleep-wake cycles. The suprachiasmatic nucleus of the hypothalamus functions as a master clock and facilitates entrainment to light (Saper et al. 2010). Relevantly, the cerebellum also contains a circadian oscillator and expresses clock genes that contribute to the regulation of circadian rhythms (Mendoza et al. 2010; Rath et al. 2012, 2014).

The cerebellum shows activity patterns related to two fundamental sleep stages: rapid eye movement (REM) and nonrapid eye movement (NREM) (for reviews, see Benarroch 2023, Canto et al. 2017, Salazar Leon & Sillitoe 2022). Both CFs and MFs show differential activity during sleep, cycling between relatively low activity during NREM sleep and high activity during REM sleep (Marchesi & Strata 1970, 1971). There are also changes in PC and CN spike rates (Mano 1970, McCarley & Hobson 1972, Zhang et al. 2020) and network dynamics that intersect with the hippocampus and the cerebral cortex across different sleep stages (Rowland et al. 2010, Torres Herraez et al. 2022, Xu et al. 2021). The latter is especially intriguing due to the implications of cerebellar function during offline memory consolidation (Geva-Sagiv & Nir 2019) and motor learning refinement (Walker et al. 2003) during sleep (for a review, see Jackson & Xu 2023).

Cerebellar delta oscillations and associated phasic sharp potentials modulate both local cerebellar very-high-frequency oscillations and distant hippocampal theta oscillations in a sleep state–dependent manner (Torres Herraez et al. 2022). Moreover, both single-unit and population activity in the cerebellum and primary motor cortex (M1) show similar fluctuating spike patterns and reciprocal fast and slow oscillations to those in the neocortex (Xu et al. 2021). Causality measures unveiled directional information flow from the neocortex to the cerebellum during slow wave sleep, whereas during spindle events, directionality reversed from the cerebellum to the thalamus and neocortex, suggesting a cerebellar contribution to neocortical sleep spindles and the shaping of sleep architecture. The changes in cerebellothalamocortical coherence during different sleep stages suggest that there are periods when the cerebellum is particularly well tuned to access and interact with thalamic and cortical regions (Xu et al. 2021). Interestingly, even though the relative spike timing between M1 and the cerebellum changes between sleep up-states and movements, the temporal relationships between M1-cerebellum pairs remain correlated in both states (Xu et al. 2022). Such findings suggest that these awake patterns of brain activity could be recapitulated during sleep, hinting at a process of offline learning consolidation. In addition, cerebellar activity during sleep aids sensory predictions and sensorimotor development. During early postnatal development in young rats, sensory-related ventrolateral and ventral-posterior thalamic activity starts to precisely mimic spontaneous twitches during REM sleep, and the precise co-occurrence of thalamic activity and movement gets refined over the course of postnatal day 12 to 20 (Dooley et al. 2021). This predictive behavior is dependent on functional cerebellar output, as pharmacological inactivation of the interposed nucleus effectively disrupts it.

Many of the monoaminergic inputs, such as cholinergic, noradrenergic, serotonergic, histaminergic, orexinergic, and dopaminergic, that influence the wake-sleep networks also impact cerebellar activity and have differential implications in local circuit plasticity and sleep-related cerebellar-dependent consolidation (for a review, see Schweighofer et al. 2004). Cerebellar activity can also modulate dopamine release in the PFC (Carta et al. 2019, Mittleman et al. 2008, Rogers et al. 2011), impinging on reward processing in the brain. Thus, to fully understand the importance of sleep stage–dependent cerebellar activity and neocortical-cerebellar interactions in memory formation and consolidation, additional research that aims to connect cerebellar intrinsic plasticity, cerebellar electrophysiology, and cerebellar structural and functional interactions with multiple extracerebellar structures during distinct sleep stages and architectural conditions will be required.

## 4. CEREBELLAR FUNCTION IN DIVERSE DISEASES

### 4.1. Neurological and Neuropsychiatric Diseases with Cerebellar Involvement

The breadth of cerebellar function and its impact on cerebellar-dependent local and global network dynamics likely contribute to guiding the specificity, complexity, and diversity of various neurological and neuropsychiatric diseases. Symptoms resulting from localized lesions in specific cerebellar regions manifest along its functional divisions, such as impaired balance and eye movement control with vestibulocerebellar dysfunction and altered gait with spinocerebellar dysfunction. Cerebrocerebellar dysfunction can lead to cerebellar cognitive affective syndrome (CCAS), also known as Schmahmann syndrome (for a review, see Schmahmann et al. 2019). According to the dysmetria of thought hypothesis, the cerebellum's primary function is to ensure accuracy and to fine-tune the coordination of motor and nonmotor processes. Therefore, cerebellar disruptions lead to the incoordination of motor and nonmotor processes that mirror each other, with the latter resulting in CCAS. CCAS is characterized by executive function deficits, spatial cognition impairments, personality changes, and linguistic difficulties. Despite these clinical observations appearing to align closely with the general functional subdivisions of the cerebellum, focal and well-isolated lesions are rare and heterogeneous. Indeed, lesion network mapping shows limited overlap in lesion locations among individuals with a common phenotype (Fox 2018). Instead, the lesioned sites are often associated with common remote networks. Furthermore, when studying a cohort of individuals with similar brain-related conditions, person-specific deviations in gray matter volume are distributed across various regions of the brain; and yet, these deviations often converge onto common circuits or networks that underlie specific brain functions (Segal et al. 2023). These studies not only offer a plausible neural substrate for heterogeneity across observable phenotypes and commonalities among specific brain functions but also provide an explanation for the impact of localized cerebellar disruptions that reverberate through distributed brain networks.

Studying developmental cerebellar circuit disruptions gives us further insights into how localized cerebellar circuits function within distributed global networks to facilitate computations for intricate behavior (for reviews, see Gill & Sillitoe 2019, Haldipur & Millen 2019, Haldipur et al. 2022, van der Heijden et al. 2021a). We focus on autism spectrum disorders (ASDs) as a model condition to briefly explore this idea. ASDs encompass a range of diverse neurodevelopmental conditions marked by deficient social interactions and core symptoms of restricted or repetitive behaviors that typically emerge in early infancy (Am. Psychiatr. Assoc. 2013). While there is a wide spectrum of etiologies and phenotypes, the cerebellum consistently emerges as a prominent site of brain pathology in individuals with ASD (Fatemi et al. 2012), with disruptions converging at PC function (for reviews, see Gill & Sillitoe 2019, van der Heijden et al. 2021a). In ASD, improper migration of PCs leads to reduced numbers of them reaching their final position (Whitney et al. 2009). Genetic evidence further links ASDs to disrupted cerebellar development, with numerous ASD-associated genes exhibiting high expression in the developing cerebellar cortex of mice (Clifford et al. 2019) and humans (Aldinger et al. 2021). Cerebellar abnormalities in both structural and functional connectivity between right crus I/II and regions of the default mode network are found in individuals with ASDs (for a review, see Olivito et al. 2023). In contrast to abrupt cerebellar lesions that result in motor deficits, social and cognitive deficits in ASDs likely stem from more widespread, potentially relatively mild but accumulative, developmental disruptions and aberrant PC function. Disrupting the development of even a single cerebellar neuronal population using conditional genetics has been shown to have a profound consequence on cerebellar neuron scaling and circuit function (Beckinghausen & Sillitoe 2019, van der Heijden et al. 2021b, Willett et al. 2019). Thus, to fully fathom the nonmotor functional consequences of altering cerebellar development, it will be critical to have a clear understanding of how different genes, cells, and circuits impact cerebellar-dependent local and global network dynamics during behavior.

### 4.2. Cerebellar Stimulation

Since cerebellar dysfunction impacts both motor and nonmotor functions, it will be crucial to develop therapies that are capable of addressing both domains. Cerebellar stimulation appears well positioned to achieve this dual benefit. Cerebellar stimulation is emerging as a promising strategy for both motor and nonmotor disorders (Baker et al. 2023, E.G. Brown et al. 2020, Cajigas et al. 2023, Farzan et al. 2013, Gatti et al. 2021, Horisawa et al. 2021, Nakamura et al. 2019, Rastogi et al. 2017, Song et al. 2020, Stroud et al. 2022, Tai & Tseng 2022). Toward this goal, significant research efforts are now beginning to extend the application of cerebellar stimulation beyond its use in motor conditions, as studies are now investigating its potential in treating psychiatric disorders such as schizophrenia (Garg et al. 2013, 2016; Hua et al. 2022), depression (D'Urso et al. 2022, Mehta et al. 2020), and obsessive-compulsive disorder (Akbari et al. 2022). Moreover, combining non-invasive methods, such as transcranial magnetic stimulation with electroencephalography, offers additional measures and insights into broader long-distance neural communication patterns in humans by allowing investigations of cortico-cerebellar connectivity in healthy and disease-related cohorts (Fernandez et al. 2020).

A key benefit to cerebellar stimulation is its potential to modulate a wide range of connected cortical and subcortical regions (for reviews, see Benussi et al. 2023, Grimaldi et al. 2014, Miterko et al. 2019, Tai & Tseng 2022, van Dun et al. 2016) and drive long-term synaptic and nonsynaptic plasticity in healthy and dysfunctional states (Cheron et al. 2013; Cooperrider et al. 2014; D'Angelo et al. 2016; Koch et al. 2014, 2019; Pauly et al. 2021; Song et al. 2020). Stimulation-driven plasticity appears to depend on LTP and the activation of *N*-methyl-d-aspartate receptors (NMDARs) since it can be blocked by NMDAR antagonists (Huang et al. 2007, Liebetanz et al. 2002). Applying deep brain stimulation (DBS) to the CN may involve the regulation of rebound burst firing, which is strongly linked to Ni^2+^-sensitive T-type Ca^2+^ channels (Alviña et al. 2009, Molineux et al. 2008), and/or the normalization (or replacement) of local circuit activity and node-specific neurotransmission (A.M. Brown et al. 2020, Miterko et al. 2021, White & Sillitoe 2017). There are also possible downstream effects on broader cerebellocortical, dentatothalamocortical, and basal ganglia networks (Baker et al. 2023, Cajigas et al. 2023, Hernandez-Martin et al. 2022, Tai & Tseng 2022), which could occur through the modulation of the release of monoamine neurotransmitters such as dopamine (Fonteneau et al. 2018) or the expression and activity of anti-inflammatory cytokines that affect neural excitability (Kumar et al. 2022). Certainly, the beneficial effects of cerebellar stimulation may be more generalized, where stimulation interferes with rather than modulates these cellular and molecular properties. The effects could also be network and behavior specific.

Precise cerebellar targeting is crucial for optimizing the benefits and minimizing the unwanted side effects of neurostimulation as the enhanced precision allows the modulation of specific neural activity in the desired pathways. Indeed, recent developments in DBS technology have allowed for more precise and flexible stimulation. Densely packed stimulation contacts can be independently activated to manipulate the electric field, honing in on pathways of interest and avoiding potential side effects (Åström et al. 2015, 2018; Lee et al. 2019). Experimental approaches in animal models are providing new insights. For example, the limitations in targeting neural pathways through conventional DBS approaches could be overcome by leveraging optogenetics. Spix et al. (2021) devised an intricate electrical stimulation technique involving burst stimulation, which facilitated population-specific neuromodulation within the external globus pallidus, yielding sustained effects in a mouse model of Parkinson's disease.

Further research is required to unravel the specific circuit mechanisms through which cerebellar stimulation impacts motor and nonmotor symptoms. This will entail understanding the intricate neural circuits and pathways involved, as well as the effects of stimulation on neuronal activity, neurotransmitter systems, and neuroplasticity across the interconnected regions. In parallel, comprehensive clinical trials and longitudinal studies are needed to continue to evaluate the long-term efficacy, durability, and safety of cerebellar brain stimulation across different disorders.

## 5. CONCLUSION

Even though the cerebellum is patterned into an exquisite and well-conserved map that consists of a relatively small number of thoroughly documented cell types, its circuitry displays remarkable diversity. As a result, the cerebellum is capable of facilitating specialized processes by segregating its functions through molecular, cellular, and local network specializations. Moreover, although the computations that drive a given behavior are represented across multiple lobules, they converge at their output to facilitate interactions with the forebrain to make rapid adaptations that meet specific demands in diverse environments. Therefore, the cerebellum can contribute to multiple complex behaviors by layering flexibility upon its fundamental modular plan. However, there are still many unknowns in the specifics of how the cerebellum achieves this. It would be useful for future studies to consider this problem using Marr's three levels of analysis: (*a*) the computational level of identifying the task that needs to be solved (i.e., pattern recognition versus control), (*b*) the algorithmic level of identifying which method to use (i.e., classification versus regression), and (*c*) the implementational level of identifying how the necessary processes will be carried out in neural tissue (i.e., a small learning capacity system versus a huge learning capacity system) (see examples in Dean & Porrill 2016, Diedrichsen et al. 2019, Kawato et al. 2021).

## Supplementary Material

Supplementary Material

## SUMMARY POINTS

1. The stereotyped cytoarchitecture of the cerebellum, its modular organization, and its regional functional diversity complement the expression of computational principles that govern its involvement in both motor and nonmotor behavior by allowing for the specialization, segregation, and distribution of its functions at the micro-, meso-, and macrolevels.
2. The flexibility of cerebellar circuits required to support a diverse range of functions is supported by specialized zones that are dedicated to specific behaviors, parallel processing, and coherent communication between brain regions. These factors enable the cerebellum to effectively engage a complex network of circuits to execute motor and nonmotor behaviors.
3. Unraveling divergent and convergent neural encoding mechanisms that underlie cerebellar adaptation, learning, and memory across motor, affect, and cognition behaviors may offer insights into whether cerebellar-dependent disorders have unique or common origins.
4. Cerebrocerebellar network–based interventions offer new and exciting therapeutic possibilities and opportunities to investigate cerebellar-dependent local and global network dynamics.

## FUTURE ISSUES

1. What is the impact of regional heterogeneity and molecular specializations within and across different modules? How do they contribute to cerebellar input and output transformations? Do distinct molecular segments within the cerebellar cortex, or even the diverse molecular subtypes of cerebellar interneurons, execute inherently different computations?
2. How do error-based signals and reward-related signals interact within local microcircuits and at downstream brain areas? Where and how do cerebellar-dependent motor, emotional, and cognitive processes converge and diverge from each other?
3. Is there a singular core computational, algorithmic, and implementational strategy that the cerebellum undertakes across various behaviors, or does it recruit multiple strategies across different levels? How are the specialization, segregation, and distribution of different functions achieved at the micro-, meso-, and macroscale levels? How is the bivalent control of behavior encoded within the anatomy, structure, and physiology of the cerebellum?
4. What determines the predominant symptomology in motor and nonmotor diseases following perturbations in cerebellar circuitry? What are the critical neural substrates and time periods that influence differential vulnerability, initiation, progression, and modulation of cerebellar-dependent disorders? How do different forms of cerebellar circuit dysfunction influence developmental and adult cerebellar circuit processing and cerebellar-forebrain interactions? Where and how would they converge and diverge across Marr's three levels of analysis?

## Abbreviations

Modules: longitudinal zones of Purkinje cells with related olivo-cortico-nuclear connections that partition the cerebellum into functional units that map onto an equivalent underlying anatomy
Lobules: structures arising from a series of fissures and folds that separate the cerebellar cortex along the anterior-posterior axis
Simple spike: the typical discharge of a Purkinje cell in the form of a single action potential, modulated in response to excitatory input from mossy fibers to granule cells
Complex spike: a low-frequency Purkinje cell profile comprising a single large spike that is followed by a series of multiple spikelets occurring in a rapid succession, which is induced by strong input from climbing fibers that originate in the inferior olive
Spatial working memory: a specific type of working memory that involves the temporary encoding, storage, and retrieval of spatial information
Coherence: the degree of synchrony or coordination in neural activity between different regions of the brain as a measure of how stable the phase relation is between two oscillations of similar frequencies
Circadian rhythm: daily 24-h cycle of physiological behaviors that are regulated by a network of cerebral clocks and peripheral oscillators
Spike rate/frequency: the number of action potentials (spikes) generated by a neuron within a specified period, typically measured in spikes per second (Hz)
Spike pattern: specific temporal arrangement (timing and sequence) of action potentials (spikes) generated by a neuron
Conditional genetics: a genetic approach that allows for the control of gene expression in specific cells and at specific developmental stages, enabling the study of a gene's function under precisely targeted and controlled conditions
Deep brain stimulation: a neurosurgical procedure that uses implanted electrodes and a pulse generator to deliver defined electrical currents to a specific brain region
Optogenetics: a technique that introduces genes encoding light-sensitive proteins into specific types of brain cells to precisely monitor and/or control their activity using light signals
