# Supplementary material for: Cerebellar Functions Beyond Movement and Learning
Source: Annu Rev Neurosci. Author manuscript; Available in PMC 2026 Jul 15. (PMC13371864; doi:10.1146/annurev-neuro-100423-104943)
Supplement: Supplementary Material [file NIHMS2184529-supplement-Supplementary_Material.pdf]

Supplemental Material: Annu. Rev. Neurosci. 2024. 47:145-166
https://doi.org/10.1146/annurev-neuro-100423-104943
Cerebellar Functions Beyond Movement and Learning
Kim, Heck, and Sillitoe

Downloaded from www.annualreviews.org. Guest (guest) IP: 128.249.96.34 On: Fri, 05 Jun 2026 20:08:57

SUPPLEMENTAL MATERIAL

CEREBELLAR ZONAL ORGANIZATION

The alternating expression of proteins and antigens, such as 5'-nucleotidase, zebrinI, zebrinII, $GABA_{B2}$ receptors, excitatory amino acid transporter 4 (EAAT4), and splice variant b of the metabotropic glutamate receptor 1, forms a robust array of parasagittal stripes that has been instrumental in understanding the intricate organization of olivo-cortico-nuclear connections and their corresponding olivonuclear and nucleo-olivary projections (see reviews Cerminara et al. 2015; Kebschull et al. 2023; Sillitoe & Joyner 2007; Sugihara 2011; Sugihara et al. 2009). For example, proprioceptive and cutaneous information from the spinocerebellar tract is received in lobules I–V and VIII/IX (Arsénio Nunes & Sotelo 1985; Brochu et al. 1990; Sillitoe et al. 2010), whereas balance and postural information from the vestibulocerebellar tract is received mainly in lobules IX and X (Jaarsma et al. 1997; Maklad & Fritzsch 2003). Moreover, zebrin-positive and zebrin-negative PCs project to distinct regions of the cerebellar nuclei that do not overlap (Chung et al. 2009; Sugihara et al. 2009), and the intrinsic properties of PCs vary across different cerebellar lobules (see reviews Cerminara et al. 2015; De Zeeuw 2021). PCs exhibit variations in the firing rate of SS and CS where distribution patterns of zebrinII and EAAT4 are associated with differences in the intrinsic firing activity and synaptic plasticity of PCs (Beekhof et al. 2021; Wadiche & Jahr 2005; Xiao et al. 2014; Zhou et al. 2014, 2015). Regional differences in the intrinsic properties of PCs and their connectivity can lead to variations in how these cells process and integrate information, resulting in specificity in function within the cerebellum (see review Cerminara et al. 2015). For example, different encoding mechanisms, such as rate coding (frequency of action potentials), temporal coding (timing of action potentials), and burst coding

(bursting of action potentials), may contribute to the varied computational capacity and efficiency in integrating and transmitting information, affecting adaptation, learning, and memory.

CEREBELLAR MODULAR ORGANIZATION

A longitudinal zone of PCs sharing similar olivo-cortico-nuclear, nucleo-olivary and reciprocal olivo-nuclear connections, and biochemical identity forms a module; some longitudinal zones, such as ones in the paravermis, can have one IO neuron innervating spatially separate regions within the broader longitudinal zone, forming multizonal microcomplexes (Apps & Garwicz 2005). Thus, longitudinal zones likely represent composite entities that contain smaller operational units, which are anatomically segregated and can function independently to control different aspects of one or more complex behaviors (Apps & Garwicz 2005).

DIFFERENCES IN LEARNING MECHANISMS

The vestibuloocular reflex (VOR) is crucial for stabilizing gaze with compensatory eye movements during head rotation and can be adapted with altering visual feedback. The VOR adaptation is under the control of upbound zebrin-positive PCs with relatively low baseline SS firing activity (50-70 Hz) that increases with contraversive head rotation in a linear fashion during the initial learning phase (De Zeeuw et al. 1995; Lisberger et al. 1994; Payne et al. 2019; Raymond & Lisberger 1998; Voges et al. 2017). When the vestibular signals coincide with head rotation, the synaptic connections between the vestibular afferents and the PCs are thought to be weakened through LTD. However, genetically blocking LTP at the PF-PC synapses leads to more pronounced impairments in VOR adaptation than blocking LTD (Gutierrez-Castellanos et al. 2017; Schonewille et al. 2010, 2011).

Downloaded from www.annualreviews.org. Guest (guest) IP: 128.249.96.34 On: Fri, 05 Jun 2026 20:08:57

Eyelid conditioning involves pairing a conditioned stimulus that normally does not evoke a reflexive eyelid response, such as a neutral tone, with an unconditioned stimulus, such as a noxious corneal air puff, which elicits an unconditioned reflexive eyelid response. This is postulated to be under the control of downbound zebrin-negative PCs with relatively high baseline SS firing rates (80-110 Hz) that decrease during the temporal interval between the conditioned and unconditioned stimuli (Halverson et al. 2015; Jirenhed & Hesslow 2011; ten Brinke et al. 2015). This SS suppression appears to be both necessary and sufficient for the expression of conditioned eyelid response as optogenetic activation or inhibition of the PCs is sufficient to eliminate or drive the conditioned response, respectively (Heiney et al. 2014; Ten Brinke et al. 2017). Moreover, concurrent blocking of LTD at PF-PC synapses and feedforward inhibition of PCs by the MLIs severely impair the conditioned response (Boele et al. 2018).

Downloaded from www.annualreviews.org. Guest (guest) IP: 128.249.96.34 On: Fri, 05 Jun 2026 20:08:57

CHALLENGES TO THE UNIVERSAL CEREBELLAR TRANSFORM

Because the cerebellum was perceived as having uniform cytoarchitecture and circuitry, it seemed reasonable to hypothesize that a conserved cerebellar computation could be generalized across functional domains. This hypothesis, known as the "universal cerebellar transform (UCT)", suggests that differences in functional roles across different cerebellar regions stem primarily from variances in afferent and efferent connectivity (see review Schmahmann et al. 2019). However, the large body of evidence of non-uniform cytoarchitecture and regional heterogeneity, encompassing factors like cell types, density, size, morphology, physiology, and regional circuit specializations, challenges this perspective (see reviews Cerminara et al. 2015; Hull & Regehr 2022). This diversity offers cerebellar processing the flexibility and specificity required to support

various functions within distinct domains. For example, MFs from diverse sources can activate a single GrC (Huang et al. 2013) or a larger pool of GrCs (DiGregorio et al. 2002), evoking random input mixing and sparse decorrelated GrC responses that can support differential inputs and increase pattern separation (Chabrol et al. 2015; Shuster et al. 2021). Moreover, contrary to the assumed monosynaptic CF input onto one PC, multibranched PCs, which receive more than one CF input, may be more common than previously thought, and interestingly many such connections are found in the posterior lobe which is associated with cognitive and limbic functions (Busch & Hansel 2023). These PCs can integrate functionally independent CF-receptive fields, giving rise to independent computational compartments that may give a computational advantage for adaptive control in a multimodal environment; however, this remains to be investigated.

Downloaded from www.annualreviews.org. Guest (guest) IP: 128.249.96.34 On: Fri, 05 Jun 2026 20:08:57

LITERATURE CITED

Apps R, Garwicz M. 2005. Anatomical and physiological foundations of cerebellar information processing. *Nat. Rev. Neurosci.* 6(4):297–311

Arsénio Nunes ML, Sotelo C. 1985. Development of the spinocerebellar system in the postnatal rat. *J. Comp. Neurol.* 237(3):291–306

Beekhof GC, Gornati SV, Canto CB, Libster AM, Schonewille M, et al. 2021. Activity of Cerebellar Nuclei Neurons Correlates with ZebrinII Identity of Their Purkinje Cell Afferents. *Cells*. 10(10):2686

Boele H-J, Peter S, Ten Brinke MM, Verdonschot L, IJpelaar ACH, et al. 2018. Impact of parallel fiber to Purkinje cell long-term depression is unmasked in absence of inhibitory input. *Sci. Adv.* 4(10):eaas9426

Brochu G, Maler L, Hawkes R. 1990. Zebrin II: a polypeptide antigen expressed selectively by Purkinje cells reveals compartments in rat and fish cerebellum. *J. Comp. Neurol.* 291(4):538–52

Busch SE, Hansel C. 2023. Climbing fiber multi-innervation of mouse Purkinje dendrites with arborization common to human. *Science*. 381(6656):420–27

Cerminara NL, Lang EJ, Sillitoe RV, Apps R. 2015. Redefining the cerebellar cortex as an assembly of non-uniform Purkinje cell microcircuits. *Nat. Rev. Neurosci.* 16(2):79–93

Chabrol FP, Arenz A, Wiechert MT, Margrie TW, DiGregorio DA. 2015. Synaptic diversity enables temporal coding of coincident multisensory inputs in single neurons. *Nat. Neurosci.* 18(5):718–27

Chung SH, Marzban H, Hawkes R. 2009. Compartmentation of the cerebellar nuclei of the mouse. *Neuroscience*. 161(1):123–38

Downloaded from www.annualreviews.org. Guest (guest) IP: 128.249.96.34 On: Fri, 05 Jun 2026 20:08:57

Downloaded from www.annualreviews.org. Guest (guest) IP: 128.249.96.34 On: Fri, 05 Jun 2026 20:08:57

De Zeeuw CI, Wylie DR, Stahl JS, Simpson JI. 1995. Phase relations of Purkinje cells in the rabbit flocculus during compensatory eye movements. *J. Neurophysiol.* 74(5):2051–64

De Zeeuw CI. 2021. Bidirectional learning in upbound and downbound microzones of the cerebellum. *Nat. Rev. Neurosci.* 22(2):92–110

DiGregorio DA, Nusser Z, Silver RA. 2002. Spillover of glutamate onto synaptic AMPA receptors enhances fast transmission at a cerebellar synapse. *Neuron*. 35(3):521–33

Gutierrez-Castellanos N, Da Silva-Matos CM, Zhou K, Canto CB, Renner MC, et al. 2017. Motor Learning Requires Purkinje Cell Synaptic Potentiation through Activation of AMPA-Receptor Subunit GluA3. *Neuron*. 93(2):409–24

Halverson HE, Khilkevich A, Mauk MD. 2015. Relating cerebellar purkinje cell activity to the timing and amplitude of conditioned eyelid responses. *J. Neurosci.* 35(20):7813–32

Heiney SA, Kim J, Augustine GJ, Medina JF. 2014. Precise control of movement kinematics by optogenetic inhibition of Purkinje cell activity. *J. Neurosci.* 34(6):2321–30

Huang C-C, Sugino K, Shima Y, Guo C, Bai S, et al. 2013. Convergence of pontine and proprioceptive streams onto multimodal cerebellar granule cells. *eLife*. 2:e00400

Hull C, Regehr WG. 2022. The Cerebellar Cortex. *Annu. Rev. Neurosci.* 45:151–75

Jaarsma D, Ruigrok TJ, Caffé R, Cozzari C, Levey AI, et al. 1997. Cholinergic innervation and receptors in the cerebellum. *Prog. Brain Res.* 114:67–96

Jirenhed D-A, Hesslow G. 2011. Time course of classically conditioned Purkinje cell response is determined by initial part of conditioned stimulus. *J. Neurosci.* 31(25):9070–74

Kebschull JM, Casoni F, Consalez GG, Goldowitz D, Hawkes R, et al. 2023. Cerebellum Lecture: the Cerebellar Nuclei-Core of the Cerebellum. *Cerebellum*

Downloaded from www.annualreviews.org. Guest (guest) IP: 128.249.96.34 On: Fri, 05 Jun 2026 20:08:57

Lisberger SG, Pavelko TA, Bronte-Stewart HM, Stone LS. 1994. Neural basis for motor learning in the vestibuloocular reflex of primates. II. Changes in the responses of horizontal gaze velocity Purkinje cells in the cerebellar flocculus and ventral paraflocculus. *J. Neurophysiol.* 72(2):954–73

Maklad A, Fritzsch B. 2003. Development of vestibular afferent projections into the hindbrain and their central targets. *Brain Res. Bull.* 60(5–6):497–510

Payne HL, French RL, Guo CC, Nguyen-Vu TB, Manninen T, Raymond JL. 2019. Cerebellar Purkinje cells control eye movements with a rapid rate code that is invariant to spike irregularity. *eLife*. 8:e37102

Raymond JL, Lisberger SG. 1998. Neural learning rules for the vestibulo-ocular reflex. *J. Neurosci.* 18(21):9112–29

Schmahmann JD, Guell X, Stoodley CJ, Halko MA. 2019. The theory and neuroscience of cerebellar cognition. *Annu. Rev. Neurosci.* 42:337–64

Schonewille M, Belmeguenai A, Koekkoek SK, Houtman SH, Boele HJ, et al. 2010. Purkinje cell-specific knockout of the protein phosphatase PP2B impairs potentiation and cerebellar motor learning. *Neuron*. 67(4):618–28

Schonewille M, Gao Z, Boele H-J, Veloz MFV, Amerika WE, et al. 2011. Reevaluating the role of LTD in cerebellar motor learning. *Neuron*. 70(1):43–50

Shuster SA, Wagner MJ, Pan-Doh N, Ren J, Grutzner SM, et al. 2021. The relationship between birth timing, circuit wiring, and physiological response properties of cerebellar granule cells. *Proc Natl Acad Sci USA*. 118(23):e2101826118

Sillitoe RV, Joyner AL. 2007. Morphology, molecular codes, and circuitry produce the three-dimensional complexity of the cerebellum. *Annu. Rev. Cell Dev. Biol.* 23:549–77

Sillitoe RV, Vogel MW, Joyner AL. 2010. Engrailed homeobox genes regulate establishment of the cerebellar afferent circuit map. *J. Neurosci.* 30(30):10015–24

Sugihara I, Fujita H, Na J, Quy PN, Li B-Y, Ikeda D. 2009. Projection of reconstructed single Purkinje cell axons in relation to the cortical and nuclear aldolase C compartments of the rat cerebellum. *J. Comp. Neurol.* 512(2):282–304

Sugihara I. 2011. Compartmentalization of the deep cerebellar nuclei based on afferent projections and aldolase C expression. *Cerebellum*. 10(3):449–63

ten Brinke MM, Boele H-J, Spanke JK, Potters J-W, Kornysheva K, et al. 2015. Evolving models of pavlovian conditioning: cerebellar cortical dynamics in awake behaving mice. *Cell Rep.* 13(9):1977–88

Ten Brinke MM, Heiney SA, Wang X, Proietti-Onori M, Boele H-J, et al. 2017. Dynamic modulation of activity in cerebellar nuclei neurons during pavlovian eyeblink conditioning in mice. *eLife*. 6:e28132

Voges K, Wu B, Post L, Schonewille M, De Zeeuw CI. 2017. Mechanisms underlying vestibulo-cerebellar motor learning in mice depend on movement direction. *J Physiol (Lond)*. 595(15):5301–26

Wadiche JI, Jahr CE. 2005. Patterned expression of Purkinje cell glutamate transporters controls synaptic plasticity. *Nat. Neurosci.* 8(10):1329–34

Xiao J, Cerminara NL, Kotsurovskyy Y, Aoki H, Burroughs A, et al. 2014. Systematic regional variations in Purkinje cell spiking patterns. *PLoS ONE*. 9(8):e105633

Zhou H, Lin Z, Voges K, Ju C, Gao Z, et al. 2014. Cerebellar modules operate at different frequencies. *eLife*. 3:e02536

Downloaded from www.annualreviews.org. Guest (guest) IP: 128.249.96.34 On: Fri, 05 Jun 2026 20:08:57

Zhou H, Voges K, Lin Z, Ju C, Schonewille M. 2015. Differential Purkinje cell simple spike activity and pausing behavior related to cerebellar modules. *J. Neurophysiol.* 113(7):2524–36

Downloaded from www.annualreviews.org. Guest (guest) IP: 128.249.96.34 On: Fri, 05 Jun 2026 20:08:57
